# Polyamine Homeostasis in Wild Type and Phenolamide Deficient *Arabidopsis thaliana* Stamens

**DOI:** 10.3389/fpls.2012.00180

**Published:** 2012-08-17

**Authors:** Christin Fellenberg, Jörg Ziegler, Vinzenz Handrick, Thomas Vogt

**Affiliations:** ^1^Department of Cell and Metabolic Biology, Leibniz Institute of Plant BiochemistryHalle (Saale), Germany; ^2^Department of Molecular Signal Processing, Leibniz Institute of Plant BiochemistryHalle (Saale), Germany

**Keywords:** *Arabidopsis*, FMOC-derivatization, hydroxycinnamic acid, phenolamides, polyamine, spermidine, stamen

## Abstract

Polyamines (PAs) like putrescine, spermidine, and spermine are ubiquitous polycationic molecules that occur in all living cells and have a role in a wide variety of biological processes. High amounts of spermidine conjugated to hydroxycinnamic acids are detected in the tryphine of *Arabidopsis thaliana* pollen grains. Tapetum localized spermidine hydroxycinnamic acid transferase (SHT) is essential for the biosynthesis of these anther specific tris-conjugated spermidine derivatives. *Sht* knockout lines show a strong reduction of hydroxycinnamic acid amides (HCAAs). The effect of HCAA-deficient anthers on the level of free PAs was measured by a new sensitive and reproducible method using 9-fluorenylmethyl chloroformate (FMOC) and fluorescence detection by HPLC. PA concentrations can be accurately determined even when very limited amounts of plant material, as in the case of *A. thaliana* stamens, are available. Analysis of free PAs in wild type stamens compared to *sht* deficient mutants and transcript levels of key PA biosynthetic genes revealed a highly controlled regulation of PA homeostasis in *A. thaliana* anthers.

## Introduction

Polyamines (PAs) like spermidine, spermine, and their diamine precursor putrescine are small aliphatic molecules commonly found in prokaryotic and eukaryotic cells. Due to their positive charges, PAs bind to macromolecules such as DNA, RNA, and proteins. In plants PAs are key players in various physiological events such as control of cell division, flowering, and senescence, and are also involved in various responses to abiotic stress such as osmotic, drought, and salt stress as well as to biotic stress, such as, microbe and pathogen interactions (Kumar et al., 1997; Walden et al., 1997; Kusano et al., 2008; Alcázar et al., 2010). In plants, a structural isomer of spermine, thermospermine, is required for stem elongation, since deficiency of thermospermine synthase (TSPMS) in *A. thaliana* resulted in dwarfism (Kakehi et al., 2008).

Depletion of other PAs may also result in growth arrest (Imai et al., 2004), whereas their excess can be cytotoxic (Tobias and Kahana, 1995). Thus, the homeostasis of PA content within a non-toxic range is a substantial challenge for the cell. Putrescine is synthesized in plants from either ornithine by ornithine decarboxylase (ODC) or from arginine by arginine decarboxylase (ADC). *A. thaliana* lacks ODC activity and therefore strongly depends on the ADC pathway (Hanfrey et al., 2001). Putrescine is the primary substrate for subsequent spermidine and spermine biosynthesis. Spermidine and spermine are synthesized from putrescine and decarboxylated SAM (dcSAM) by spermidine synthase (SPDS) and spermine synthase (SPMS), respectively; dcSAM is produced from *S*-Adenosyl-l-methionine by SAM decarboxylase. The positional isomer of spermine, thermospermine, is synthesized from spermidine by TSPMS (Fuell et al., 2010). Alternatively, PAs can be further metabolized into alkaloids (Biastoff et al., 2009) or be conjugated to hydroxycinnamic acids (Bassard et al., 2010).

**Figure 1:**
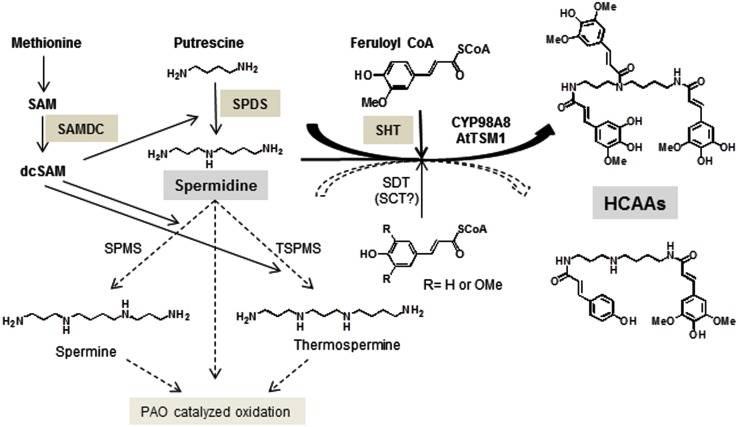
**Key steps in the biosynthetic pathway of HCAAs in *Arabidopsis* stamens**. Relevant enzymes SAMDC, SPDS, and SHT as well as the major polyamine, spermidine are specifically marked. The presence of spermidine disinapoyl transferase (SDT) transcript levels has been confirmed in this report.

Several mechanisms are employed to achieve homeostasis of intracellular PA levels. Transcriptional and translational regulation of ADC, ODC, and SAMDC was observed with a rapid turnover of the enzymes (Tabor and Tabor, 1984). PAs can be also degraded by di- or PA oxidases, DAO, and PAO (Moschou et al., 2008) or can be conjugated to phenolics (Martin-Tanguy, 1985) and last but not least, PAs can be the sequestered by transport to vacuoles or extracellular compartments (Martin-Tanguy, 2001). A simplified version of PA metabolism in *A. thaliana* anthers is illustrated in Figure 1.

Hydroxycinnamic acid amides (HCAAs) are described from reproductive organs of many plant species (Meurer et al., 1988) and only recently they have been shown to occur also in *A. thaliana* flowers, seeds, and leaves (Böttcher et al., 2008; Fellenberg et al., 2008; Luo et al., 2009; Muroi et al., 2009). While bis-conjugated derivatives, e.g., in leaves may play a role in plant defense (Onkokesung et al., 2012) tris-substituted HCAAs appear to be restricted to reproductive organs and are specifically deposited on the surface of pollen grains (Fellenberg et al., 2008; Handrick et al., 2010). They are synthesized in the tapetum, which also serves as a temporary storage for a wide range of compounds, such as sugars, carotenoids, flavonoids, fatty acids, and yet unidentified sporopollenin precursors (Hsieh and Huang, 2007; Ariizumi and Toriyama, 2011). Upon programmed cell death of the tapetum the compounds are released and accumulate on the exine of maturing pollen grains. Very recently, genes and enzymes participating in the biosynthesis of HCAAs within the tapetum were identified. The essential acyl transfer is performed by a BAHD-like hydroxycinnamate (HCA) acyltransferase, spermidine hydroxycinnamoyl transferase (SHT; Fellenberg et al., 2009, 2012; Grienenberger et al., 2009; Matsuno et al., 2009). The resulting conjugates are subsequently modified by two cytochrome P450 monooxygenases (CYP98A8, CYP98A9) and by AtTSM1, a cation-dependent *O*-methyltransferase (Fellenberg et al., 2008; Matsuno et al., 2009). Specifically *sht* knockout lines show drastic reduction of HCAAs in *A. thaliana* anthers and pollen grains.

To determine if this massive loss of conjugated compounds has any effect on spermidine metabolism and PA levels, a sensitive method was required to compare the total PA content in *A. thaliana* stamens of wild type and mutant plants. Reagents most commonly used for detection and quantification of PAs include dansyl chloride, benzoyl chloride, *O*-phthalaldehyde (OPA), and 9-fluorenylmethyl chloroformate (FMOC-Cl). The application of dansyl chloride (5-dimethylaminonaphthalene-1-sulfonyl chloride) and benzoyl chloride for quantification of PAs is well established and widely used in plant science and food technologies (Bouchereau et al., 2000). But both methods have some disadvantages as the non-specific reagents react not only with primary and secondary amino groups but also with phenolics, aliphatic alcohols, and some sugars (Bartók et al., 1992). Furthermore, in both cases sample preparation appears time consuming. Compared to dansylation and benzoylation the application of OPA is more sensitive because it reacts specifically with primary amino groups within seconds, but the formed OPA derivatives have a limited stability (Skaaden and Greibrokk, 1982). In contrast, FMOC-Cl has been used as a protective agent for amino groups in peptide synthesis and as a fluorescent derivatizing agent for the determination of amino acids (Carpino and Han, 1972; Einarsson et al., 1983). Due to their selective reactivity toward primary and secondary amino groups, FMOC-Cl can also be used for determination of PAs. The resulting FMOC-Cl derivatized PAs are stable and detection is sensitive (Yun and Zhang, 1987; Bartók et al., 1992; Huhn et al., 1995).

To monitor PA metabolism in HCAA-depleted anthers, a significantly improved and sensitive FMOC-Cl based method for PA quantification from *A. thaliana* wild type and *sht* anthers and pollen grains was developed and ratios of bound versus unbound spermidine levels were determined. Additionally, the contribution of key players in HCAA-formation, such as SPDS1 and SAMDC1, to PA levels and phenolic profile were also characterized supported by transcript profiles of the PA biosynthesis relevant genes.

## Materials and Methods

### Plant material

Wild type *A. thaliana* ecotype Columbia 1092 and all knockout mutants SALK_055511c for the *At2g19070* gene encoding SHT; the *At3g02470* gene encoding SAMDC1 and the *At1g23280* gene encoding SPDS1 were obtained from the European *Arabidopsis* Stock Center (Nottingham, UK) and homozygous mutant lines were obtained as described in detail (Fellenberg et al., 2009) or selected by PCR. T-DNA insertion was confirmed by DNA amplification using the left T-DNA border-specific primer LBa1 (5′-TGGTTCACGTAGTGGGCCATCG-3′) and the gene specific primer SAMDC1-for (5′-GGCCTTATCTGCAATCGGTTTC-3′) for the SALK_020362 line. For SPDS1 insertion line GK709C06, the T-DNA insertion was identified by PCR using a T-DNA specific primer GK08409 (5′-ATATTGACCATCATACTCATTGC-3′) and a gene specific primer SPDS1-for (5′-CAGGAGAGGCACACTCATTG-3′). Plants were grown in fully climatized greenhouses at 22°C (day) and 18°C (night) under long day conditions, with a 16-h light/8-h dark cycle. Constant light intensity was provided by SGR-K 140 lamps equipped with SON-T AGRO 400 bulbs (Philips). Flower buds, leaves, stems, and siliques were harvested from 6-week-old adult plants and 50 stamens per sample were dissected from flower buds at developmental stage 11 or 12 (Smyth et al., 1990). *A. thaliana* pollen grains were harvested and purified according to established methods (Johnson-Brosseau and McCormick, 2004; Handrick et al., 2010). Plant material was pulverized with mortar and pestle under liquid nitrogen whereas stamens and pollen grains were homogenized using a CryoMill (Retsch, Haan, Germany) and finally stored at −80°C until use.

### Extraction and derivatization of polyamines

To extract PAs, 25 mg of ground tissue, 50 stamens or 10 mg pollen were transferred to 500, 100, and 200 μl, respectively, of buffer containing (20% v/v methanol, 200 mM NaCl, 10 mM Kpi, pH 6.0), incubated for 10 min in a Sonorex Super RK 510 ultrasonic bath (Bandelin Electronic, Berlin, Germany) and centrifuged for 15 min at 4°C and 18,000 × *g*. In case of flower buds and pollen grains the remaining pellet was re-extracted once more with the same volume of extraction buffer and both supernatants were combined. For quantification via standard addition, each supernatant was aliquoted into 20 μl portions and spiked with 5 μl PA standard mixture containing putrescine, diaminoheptane (additional internal standard), spermidine, and spermine (Sigma–Aldrich, Taufkirchen, Germany) with concentrations ranging from 0 to 70 μM. Subsequently 50 μl borate buffer (0.5 M boric acid solution adjusted to pH 7.9 with NaOH) and 100 μl 3 mM FMOC in acetone (Fluka, Buchs, Switzerland) were added and 5 μl of this mix subsequently analyzed by HPLC analysis.

### Analysis of polyamines by HPLC

The derivatized PAs were separated on a Lichrospher 100 RP-18 column (5 μm, 125-4 mm; Merck, Darmstadt, Germany) using a HPLC1200 Series system (Agilent, Waldbronn, Germany) attached to a fluorescence detector (excitation wavelength 265 nm, emission wavelength 320 nm). Eluent A (water) and B (acetonitrile) both contained 0.2% (v/v) acetic acid. Elution was performed with a linear gradient from 65 to 98% eluent B in A within 20 min. The flow rate and column temperature were set to 1 ml/min and 30°C.

### Calculations of the HCAA content

The molar extinction coefficients of 5-hydroxyferulic acid and sinapic acid were calculated according to the Lambert–Beer law by measuring different concentrations (10–200 μM) photometrically at the absorption maximum of 318 and 320 nm, respectively. 5-hydroxyferulic acid HCAAs were then measured by RP-HPLC on a 12.5 cm, 4 mm id 5 μm Nucleosil C18-column (Macherey-Nagel, Düren, Germany) at a flow rate of 1 ml/min with a gradient from 10% (v/v) B (acetonitrile) in A (water, 0.5% phosphoric acid) to 50% (v/v) B in A within 10 min. The amount of the most prominent HCAA *N*_1_,*N*_10_-bis-(5-hydroxyferuloyl)-*N*_5_-sinapoyl-spermidine was deduced from the extinction coefficient of 5-hydroxyferulic acid taking three phenolic moieties into account and assuming no interference of the individual phenolic residues within a single HCAA-molecule. The extinction coefficient of sinapic acid at 320 nm is virtually identical to 5-hydroxyferulic acid and at 318 nm only marginally lower.

### qPCR and transcript analysis

For transcript analysis 200 anthers of *A. thaliana* Columbia 1092 wild type and homozygous *sht* knockout lines were pooled and analyzed according to Fellenberg et al. (2012). Briefly, RNA was extracted by a standard phenol/chloroform protocol, reverse transcribed using Superscript^®^ reverse transcriptase (Invitrogen), and qPCR was performed using SYBR^®^-green qPCR mastermix (Applied Biosystems). The small subunit of phosphatase 2A served as a reference gene. The list of relevant primers is supplied in Table 1.

**Table 1:** **Detection limits of FMOC-derivatized polyamines**.

Compound	Quantification limit LOQ [fmol]	Detection limit LOD [fmol]
Putrescine	55	27
Spermidine	55	27
Spermine	55	14
Diaminoheptane	110	55

*Values represent minimum amounts detectable after HPLC and florescence detection per injection*.

## Results

### Determination of polyamines – method development

**Figure 2:**
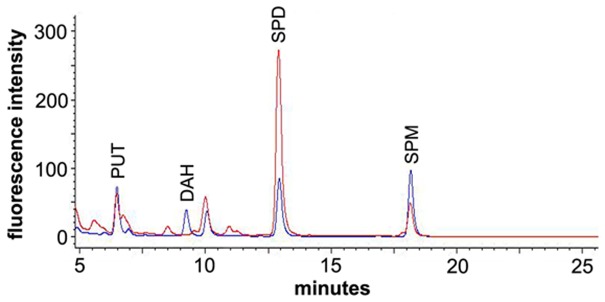
**Section of a chromatogram overlay for a PA standard mixture (blue) and a flower bud extract (red) after derivatization with FMOC-Cl**. The standard mixture contained 10 pmol each of putrescine (PUT), diaminoheptane (DAH), spermidine (SPD), and spermine (SPM).

**Figure A1:**
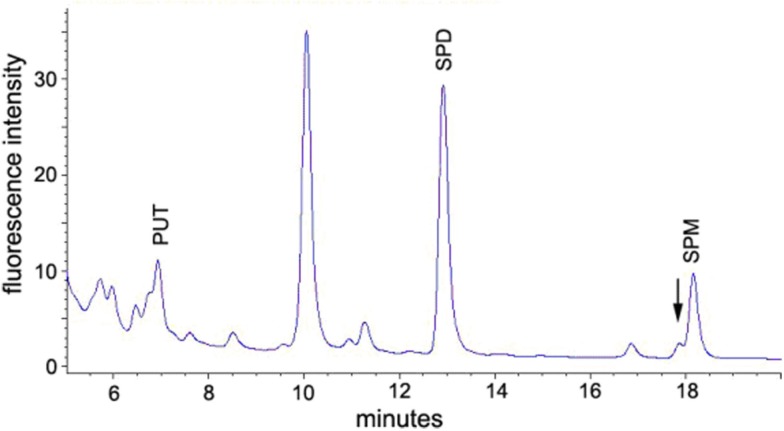
***A. thaliana* stem extract derivatized with FMOC-Cl**. The pattern of elution is identical to the flower bud extract (Figure 2). The large unmarked peak is an derivatization artifact. Put, putrescine; spd, spermidine; spm, spermine. Thermospermine most likely elutes as a left shoulder of spermidine. Notice the different scale compared to Figure 2.

After derivatization, putrescine, diaminoheptane, spermidine, and spermine could be clearly separated under the conditions used (see Materials and Methods). Putrescine and diaminoheptane were detected at 6.4 and 9.3 min retention time, respectively, while spermidine was detected at 12.9 min, spermine at 18.2 min. Figure 2 (blue line) shows a typical FLD-chromatogram obtained from a 10 pmol standard mixture of FMOC-PA derivatives. The chromatogram of a flower bud extract (red line) after derivatization with FMOC showed peaks with the same retention time excluding diaminoheptane, which was used as an internal standard and is no naturally occurring PA. The highly reproducible retention times of PA standards allowed the preliminary identification of the co-migrating peaks in plant extracts. Additional spiking with known amounts of particular standards, and increased areas of those specific peaks, confirmed the nature of compounds investigated. All PAs can be distinguished clearly using the separation conditions described above. The peak occurring after diaminoheptane (10 min) is not a result of derivatized PAs but a methodical artifact, since it also appears in samples not containing any PAs. The minor shoulder in front of spermine is likely thermospermine. However, no standard was available for this compound. Extracts of stem tissue clearly indicate a more prominent peak at 17.9 min slightly before spermine (Figure A1 in Appendix), consistent with data of benzoylated thermospermine eluting slightly earlier than the corresponding spermine derivative (Naka et al., 2010). The elution profile in this report is optimized for spermidine detection. A better separation of spermine and thermospermidine might be achieved by using longer RP-columns, a column with smaller particle size or optimization of gradient conditions. Advantageously, the relative hydrophobicity of all FMOC-PAs allows for a quite apolar gradient minimizing the interference with other endogenous, usually more polar, derivatized compounds, i.e., amino acids.

**Figure 3:**
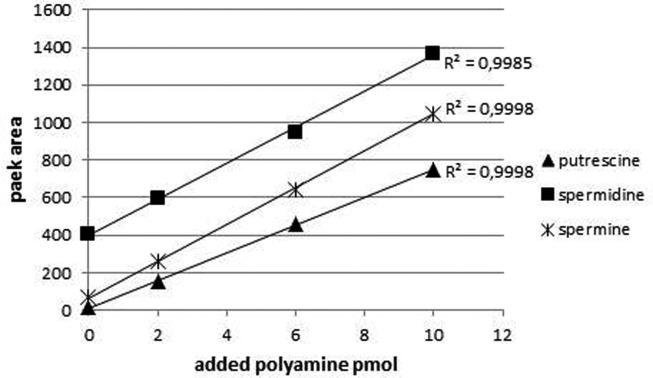
**Typical calibration curves for the PAs of interest quantified by standard addition method**. Leaf extracts were supplemented with different amounts of PAs. The curves are linear and the correlation coefficient for all three compounds is above 0.999. The ordinate indicates the fluorescence intensity: excitation at 265 nm; emission at 320 nm.

To quantify PAs in plant tissues the method of standard addition was used. For methods not relying on mass spectrometry, this procedure represents the most-effective way to compensate for the adverse influence of the matrix on method performances (Trufelli et al., 2011). After extracting the PAs from the tissue, extracts were partitioned into four aliquots and to each of them an equimolar mixture of PA standards was added and after derivatization analyzed by HPLC (final amounts on column: 0, 2, 6, and 10 pmol). Figure 3 shows an example of a standard addition plot in leaf extracts. The concentration of each standard was plotted against the peak area resulting in a linear fit with correlation coefficients between 0.998 and 0.999. The intercept of abscissa and the linear regression line was used to calculate the internal amount of PAs within the original extract. Besides putrescine, spermidine, and spermine, also diaminoheptane was added to the samples and the peak area was plotted against the added amount (data not shown). Each sample resulted in a linear regression line with virtually identical slopes (42.9 ± 1.3) indicating the comparable derivatization efficiency in every extract and stability of the FMOC-PAs during measurement time.

### Monitoring the PA content in *A. thaliana* organs

**Figure 4:**
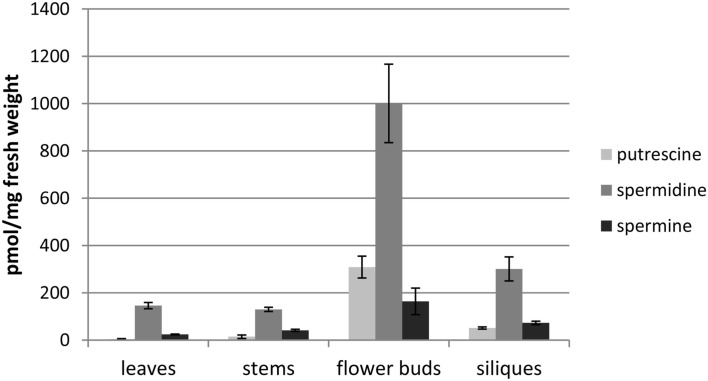
**PA content of various organs in *A. thaliana***. Mature rosette leaves, stems, flower buds, and siliques were harvested from 6-weeks-old plants and the PA content was analyzed. Mean values and ± standard deviations (*n* = 3) are shown.

The established FLD-HPLC method was applied to determine the PA content in extracts of four different organs of *A. thaliana*. Three replicates of each tissue sample were extracted, analyzed, and the PA content was calculated as described above. To be sure that all PAs were extracted the remaining pellet was re-extracted once more with the same volume of extraction buffer and both supernatants were analyzed as described above. Due to the hydrophobic and viscous properties of the pollen tryphine or the relative large amounts of PAs flower buds and pollen grains needed this second extraction. About 20% of the total PA amount was extracted with this second step. Leaves, stems, and siliques showed only negligible amounts of PAs in the remaining pellet (always less than 5%). First and second extraction of pollen and flower buds were always combined for final quantification. HPLC chromatograms showed comparable qualitative composition of the three major PAs in all four samples but revealed substantial quantitative differences. The highest levels of PAs were detected in extracts of flower buds (about 1,500 pmol/mg) followed by siliques (about 400 pmol/mg), stems (about 200 pmol/mg), and leaves (about 200 pmol/mg; Figure 4). The most prominent PA in all tissues was spermidine with around 1,000 pmol per mg fresh weight in flower buds. In stems and leaves levels were lower, about 150 pmol/mg, whereas up to 300 pmol/mg were detected in green siliques. Those observations are consistent with previously published PA levels in *A. thaliana* organs (Naka et al., 2010) and allowed to conclude that PA extraction, FMOC-Cl-derivatization, and quantification by HPLC is reliable.

### PA content in *A. thaliana* stamens

**Figure 5:**
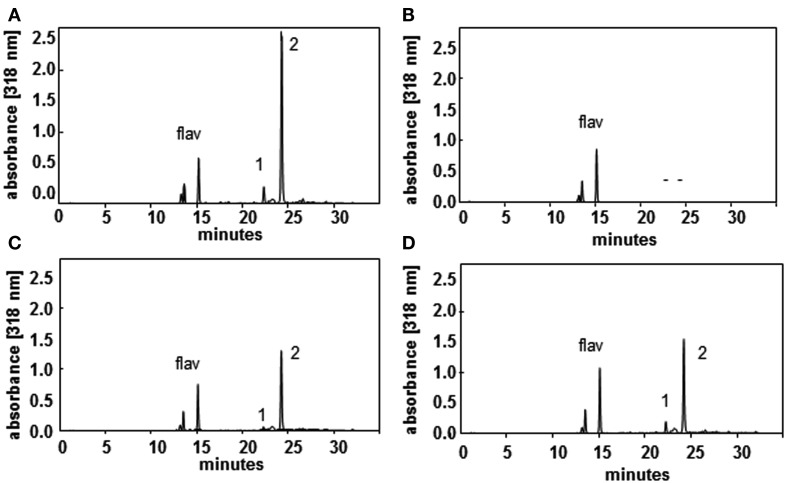
**HPLC profiles of wild type (A), *sht* (B), *spds1* (C), and *samdc1* (D) pollen recorded at 318 nm illustrate the effects on HCAA-formation in the mutants**. Major HCAAs (*N*_1_,*N*_5_,*N*_10_-tris-5-hydroxyferuloyl spermidine, (1) and *N*_1_,*N*_10_-bis-(5-hydroxyferuloyl)-*N*_5_-sinapoyl-spermidine and (2) as well as flavonoids (flav) are marked.

**Figure 6:**
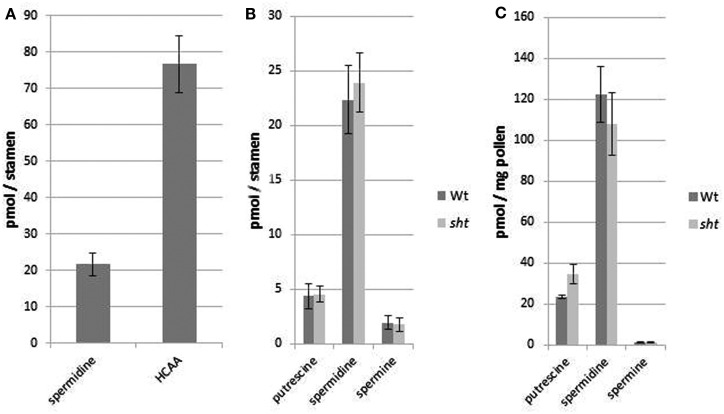
**Ratio of free and bound spermidine in *A. thaliana* wild type stamen (A) and amounts of all three PAs in stamen of wild type and *sht* stamens (B) and pollen grains (C)**. **(A)** The quantity of the single most prominent HCAA *N*_1_,*N*_10_-bis-(5-hydroxyferuloyl)-*N*_5_-sinapoyl-spermidine was compared to the amount of free spermidine in the same organs. **(B)** PA content in stamen of *A. thaliana* wild type and *sht* mutant plants. **(C)** Total polyamine levels on purified *Arabidopsis* pollen grains from wild type and *sht* plants. Mean values ± standard deviations (*n* = 3) are shown.

After developing a precise and reproducible method for quantification of PAs in plant tissue we were interested in the question, whether the lack of spermidine conjugates in *sht* knockout mutants result in a significant change, specifically an increase in PA levels in stamens. In this organ the most prominent PA, spermidine, also occurs conjugated to hydroxycinnamic acids. Tris-substituted HCAAs, which comprise the vast majority of the overall HCAAs are completely absent in *sht* knockout mutants (Handrick et al., 2010). Due to the diverse pool of different minor mono- and bis-HCAAs synthesized in the tapetum and localized on the pollen surface (Handrick et al., 2010) and the unavailability of standards for quantification, an exact calculation of the total HCAA amount was only applied for the two most prominent tris-5-hydroxyferulic acid based conjugated compounds clearly visible on HPLC chromatograms (Figure 5). To compare the ratio of free spermidine and HCAAs, the concentration of the unbound spermidine was quantified as described above whereas the amount of HCAAs was calculated based on absorbance measurements and HPLC analysis. It was assumed that all three hydroxycinnamoyl moieties contribute equally to the absorbance of *N*_1_,*N*_5_,*N*_10_-tris-5-hydroxyferuloyl and *N*_1_,*N*_10_-bis-(5-hydroxyferuloyl)-*N*_5_-sinapoyl-spermidine, respectively. The molar absorption coefficient of 5-hydroxyferulic acid and sinapic acid was determined as ε = 17,000 L mol^−1^cm^−1^ at 318 nm in 80% MeOH. Based on these calculations, the quantities and the ratio of free and HCA-bound spermidine are shown in Figure 6. Concentrations were determined on a pmol/stamen basis. 50 stamens (average weight 13 ± 1 μg/stamen) were used for each sample preparation. About 22 pmol of free spermidine was observed on a per stamen basis, equivalent to 1.7 nmol/mg, compared to a fourfold higher amount of conjugated *N*_1_,*N*_10_-bis-(5-hydroxyferuloyl)-*N*_5_-sinapoyl-spermidine (76 pmol/stamen; Figure 6A). Based on the single most prominent HCAA, the actual total amount of all HCA-bound spemidine is considerably higher than levels of free spermidine. No significant difference could be detected for free PAs levels in stamens of wild type and *sht* plants (Figure 6B). This observation suggests that the level of free PAs, specifically spermidine in stamen appears tightly controlled.

Free and HCA-conjugated spermidine levels of isolated pollen grains of wild type and *sht* lines were also measured (Figure 6C). The levels of free PAs on pollen grains were 10-fold lower compared to stamens (120 pmol/mg) on a per weight basis. As in the case of whole stamens, no differences between wild type versus *sht* plants are apparent. The total amount of the major HCAA-conjugate, *N*_1_,*N*_10_-bis-(5-hydroxyferuloyl)-*N*_5_-sinapoyl-spermidine on MeOH-washed pollen grains was calculated at 6.5 nmol/mg pollen grains. This indicates that the soluble part of the pollen exine, the tryphine is a preferential accumulation site of soluble HCAAs. In conclusion, conjugated spermidine levels on pollen grains exceed the level of free spermidine by two orders of magnitude suggesting a drastic and effective mechanism to shut down spermidine production in *sht* knockouts.

### Transcript levels of genes encoding key spermidine biosynthetic steps

**Figure 7:**
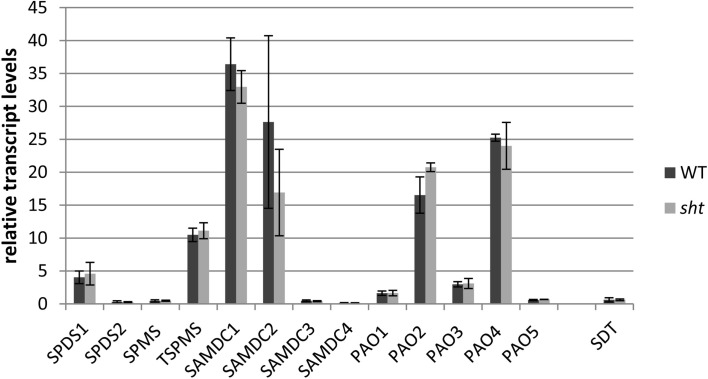
**Relative transcript levels of key polyamine pathway genes in isolated anthers of *A. thaliana* wild type compared to *sht* plants**. Corresponding gene names and primers used can be found in Table 2. Pp2A was used as a reference gene.

**Figure 8:**
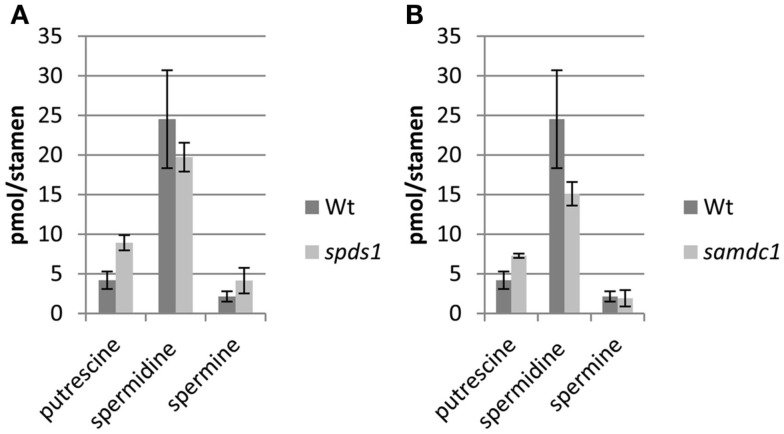
**PA levels in stamen of *A. thaliana samdc1* (A) and *spds1* (B) mutants compared to wild type**.

In an effort to determine potential regulatory steps in spermidine biosynthesis in wild type and *sht* plants, transcript levels of all genes encoding decisive steps in PA biosynthesis and degradation were measured in both accessions (Figure 7). These genes included SAMDC, SPDS, SPMS, TSPMS, PA-oxidizing enzymes, and finally SDT, the second BAHD-like transferase which showed considerable expression in stamens. These genes including primers are listed in Table 2. Initially all SAMDC and SPDS lines were also analyzed with respect to HCAA-profiles. Among the six lines investigated, only SAMDC1 and SPDS1 knockout mutants showed a marked decrease in HCAA-formation (Figure 5). When PA levels were analyzed in SPDS1 mutants, the amount of putrescine was significantly elevated, while spermidine and spermine levels appeared rather constant (Figure 8). The data are consistent with the accumulation of the SPDS1 substrate putrescine, lack of SAMDC1 results in a significant reduction of spermidine levels while spermine amounts appear virtually constant (Figure 8) suggesting that either SAMDC2, SAMDC3, and SAMDC4 completely compensate for the loss of SAMDC1. In case of SPDS, encoded by two alleles in *A. thaliana*, the *SPDS1* gene encoded by the locus *At1g23820* is 10-fold higher expressed in anthers than *SPDS2*. Our data showed that *spds1* plants also display a reduction in HCAA-accumulation (see Figure 5). This observation confirms that SPDS1 rather than SPDS2 contributes to HCAA-formation in anthers. Transcript levels of the corresponding gene in *sht* plants compared to wild type plants appear virtually unchanged (Figure 7). The same holds true for *SPDS2*. Transcript levels of all four copies of SAMDC were also measured in stamens of both lines. Compared to *SAMDC3* and *SAMDC4* only *SAMDC1* and *SAMDC2* displayed considerable expression in anthers. Transcript formation of *SAMDC2* in the *sht* line appeared slightly, but not significantly (*p* = 0.147) reduced when compared to wild type plants. However, only plants deficient in *SAMDC1* encoded by the allele *At3g02470*, but not *samdc2* lines showed a considerable effect on HCAA-formation (Figure 5), again favoring this allele to be involved in HCAA-biosynthesis. Transcript levels of the genes encoding SPMS and TSPMS appear unchanged in wild type and *sht* mutants the same holds true for the transcript levels of all alleles encoding PA oxidase genes. Although this may not be unexpected, the high transcript abundance of TSPMS, compared to the low metabolite level is puzzling. For completeness we also measured transcript levels of two additional bis-hydroxycinnamoyl-transferases already described specifically from *A. thaliana* seeds and roots by Luo et al. (2009). Although transcript levels of the disinapoyl transferase (SDT) in wild type and *sht* plants could be measured and did not differ between both plants, a reliable estimation of transcript levels of the coumaroyl transferase (sct) was unsuccessful in both cases, as we could only detect the transcript at the detection limits.

**Table 2:** **List of relevant polyamine biosynthetic genes in *A. thaliana* analyzed by transcript accumulation**.

Gene		Annotation	Primer sequence (5′ → 3′)
*S*-adenosyl-l-methionine decarboxylase (SAMDC)	SAMCD1	*At3g02470*	for TTGAAGTGTGAATGGAGCAG
			rev GATTCCCTCGTCCTTCTCG
	SAMDC2	*At5g15950*	for GGAAGGTATTGTGGTTCTCC
			rev CTATTCCTTCTCGTCCTCGC
	SAMDC3	*At3g25570*	for GGTATTGGCGTCTGATTGTG
			rev CCAAGTCCAAGCTCCTCTAG
	SAMDC4	*At5g18930*	for GAGCCGTCTTATGGATGAG
			rev CTATTTCCGACGAGGCGT
Spermidine synthase (SPDS)	SPDS1	*At1g23820*	for CTCGGAGATATTCACCACAG
			rev CTGATCTCCGTTCTCCGTCT
	SPDS2	*At1g70310*	for ACTGATTTGCCCGTGAAGAG
			rev GTTCTCTGTTTCCATGGCGC
Spermine synthase (SPMS)	SPMS	*At5g53120*	for TTCTTCAGATCCCGTAGGTC
			rev CTCTAGCCAGTGTCTCGAA
Thermospermine synthase (TSPMS/ACL5)	TSPMS/ACL5	*At5g19530*	for GCTCCTTCTTTCGTCTCTG
			rev CAGTCTCCTTCTCTAGCGC
Polyamine oxidase (PAO)	PAO1	*At5g13700*	for ACCCGGGCTCTAACATTC
			rev GATTGAGCTTCAACGCGC
	PAO2	*At2g43020*	for TTCTGGAGCGGTATGGTG
			rev GAAGAGGTACAGAGGCAGG
	PAO3	*At3g59050*	for GAGACTGAGAGTGCCATTG
			rev GAATAAGCACCGTGCACTG
	PAO4	*At1g65840*	for CAGGGAATCTAGCACAAGAC
			rev CACTAGATATTGAGCCGGGT
	PAO5	*At4g29720*	for GAAGAACCGCGACCATTAC
			rev GTTTCTCAAGCTCGAGAGC
Spermidine disinapoyl transferase (SDT)	SDT	*At2g23510*	for TATTGGGATTTTCGGATCG
			rev CCATATCCGATTCCAGCCTAGA

*Gene annotation was performed according to Ge et al. (2006) and Hanzawa et al. (2002). For (forward) and Rev (reverse) primer sequences are included*.

In summary, transcript levels of key PA biosynthetic and degrading enzymes appear not critical for constant PA levels in anthers, suggesting a feedback regulation at post-transcriptional, translational, or post-translational levels. No spermidine conjugates other than HCA-conjugated forms (like spermine, thermospermine, or tyramine) were detected by targeted mass spectrometry during prior investigations (Handrick et al., 2010) and have not been reported in the literature by others from *A. thaliana* to the best of our knowledge which precludes alternative metabolic routing.

## Discussion

In this study PA levels in *A. thaliana* wild type and HCAA-knockout lines were compared and an effective HPLC analysis of FMOC-PA derivatives using fluorescence detection was developed. In order to determine the best extraction for total PA recovery from plant tissue, multiple extractions on the same sample was performed with different extraction buffers. Twenty percent MeOH containing 200 mM NaCl and 10 mM Kpi pH 6.0 was selected to be the best solvent for PA extraction followed by direct derivatization with FMOC-Cl. Surprisingly, the application of the widely used perchloric acid as a solvent for PA extraction (Bouchereau et al., 2000) resulted in lower fluorescence intensities (data not shown). The derivatization of amines with FMOC-Cl appears optimal under basic conditions (pH 7.8) whereas at very low pH values a protonation of PAs may prevent complete derivatization (Huhn et al., 1995). Lower signals after perchloric acid extraction would then be explained by lower derivatization efficiencies rather than lower extraction yields. To remove the positively charged PAs from plant tissue involving ionic interactions, a medium salt containing buffer at physiological pH is preferable and directly compatible with the subsequent FMOC-Cl based derivatization.

Polyamine levels in different organs of *A. thaliana* can be rapidly and reproducibly quantified by this method. Putrescine, spermidine, and spermine are present in all analyzed organs of the plants but clearly accumulated in flower buds, consistent with previous reports (Kakkar and Rai, 1993). Compared to the sexual organs, PA levels were rather low in siliques, leaves, and stems. Analysis of benzoyl PAs by Urano et al. (2003) and Naka et al. (2010) in *A. thaliana* organs revealed a similar distribution pattern and comparable quantities. Thermospermine is visible as a small shoulder in front of spermine. As this study focused on the determination of spermidine, we did not apply the vast majority of opportunities in order to improve the resolution of spermine/thermospermine which should be achievable by several approaches. Nevertheless, these results suggest that the developed method is applicable to quantify PAs in plant tissue and our results corroborate previous results which were obtained with established methods. The most advantageous improvement of this method for PA research is its adaptability to very small amount of starting plant material. Previously described PA quantification methods require a minimum of plant material of 50–100 mg (Kamada-Nobusada et al., 2008; Sánchez-López et al., 2009) whereas this study now shows that less than 1 mg is sufficient enabling the determination of PA levels in very small samples with high accuracy. Compared to tobacco stamens with reported 3.4 nmol/anther (0.5 μg/anther; Chibi et al., 1994) the spermidine levels in *A. thaliana* stamens can be measured with two orders of magnitude lower sensitivity (∼20 pmol/stamen). When the different size and mass of both types of stamens are considered, the amounts on a fresh weight basis appear similar.

Comparison of the three common plant PAs, putrescine, spermidine, and spermidine in *A. thaliana* wild type stamen and a HCAA biosynthetic mutant, lacking the conjugation of HCAs toward spermidine, showed no differences in PA levels. The *sht* knockout lead to a drastic reduction in HCA-bound spermidine levels and a significant increase of free spermidine could have been expected within *sht* anthers. Instead, neither the levels of spermidine, nor the direct spermidine precursor putrescine and the amounts of the next higher PA, spermine are increased. This indicates a tight and highly controlled regulation of intracellular titers of free PAs. Apparently, PA levels in stamens are not controlled at the transcript levels of key biosynthetic enzymes. Alternative regulatory mechanisms may include *de novo* synthesis, degradation, and transport. Removal of cytotoxic over-accumulating PAs within the *sht* line by subcellular compartmentalization to the vacuole, which is one known mechanism for regulating cytosolic PA levels (Pistocchi et al., 1988), can be excluded since the whole stamen was used for PA determination. The detection of PAs in xylem and phloem sap of various plants indicates that there is PA translocation to other organs (Antognoni et al., 1998; Ohe et al., 2005). Actually, very little is known about a possible transport of PAs between plant tissues. Recently, a rice PA uptake transporter (OsPUT1) has been identified preferentially transporting spermidine (Mulangi et al., 2012) and is postulated to participate in phloem loading of PAs. It is possible that PAs in the *sht* mutants are transported out of tapetal cells into other plant organs, but to date no such PA transporters are known in *A. thaliana*.

Anthers of *sht* mutants showed no significant change on the transcriptional level for both *SPDS* and all four *SAMDC* genes compared to wild type, thus regulation of PA levels via transcriptional feedback control, as recently shown for flavonol biosynthesis (Yin et al., 2012) appears unlikely. In the latter case elimination of the final glycosyltransferase lead to a repression of the complete flavonol biosynthetic pathway, illustrating efficient transcriptional down-regulation of upstream genes like chalcone synthase and phenylalanine ammonia lyase. Only knockouts of *SAMDC1* and *SPDS1* reduce the accumulation of HCAA-conjugates in anthers. Among these, SAMDC mRNAs express 5′ leader sequences which contain a highly conserved pair of upstream open reading frames (uORFs) that overlap by a single base (Franceschetti et al., 2001). The small uORF-encoded peptide is responsible for constitutively repressing downstream translation of the SAMDC proenzyme ORF under conditions of excess PA concentration, whereas the tiny uORF is required for induced translation of the main ORF during conditions of low PA concentration (Hanfrey et al., 2005). This important translational regulation of *SAMDC* mRNA to maintain plant PA homeostasis could be responsible for the unchanged PA levels within the *sht*. Assuming that flux of spermidine into HCAAs is (partly) blocked, an overproduction of spermidine would be avoided by repressing *SAMDC1* translation within the tapetum. An alternative, mechanism involving antizyme-mediated degradation of ODC when spermidine levels rise is known from yeast and animals (Kurian et al., 2011). However, ODC does not exist in *A. thaliana* and a similar mechanism for ADC in plants is questionable although not impossible.

Alternatively, cellular PA contents could be also controlled by catabolic pathways. Therefore degradation of accumulating spermidine via enzymatic PA oxidation appears possible to keep PA levels in *sht* plants similar as in wild type stamens. PA oxidase (PAO) and diamino oxidase are involved in PA catabolism. PAOs, using FAD as cofactor and O_2_ as electron donor are able to catalyze the oxidative deamination of secondary amino groups of spermidine and spermine, producing the corresponding aldehyde, H_2_O_2_, and 1,3-diaminopropane, back-converting spermine to spermidine and spermidine to putrescine (Moschou et al., 2008). However, no changes in the level of putrescine have been observed. Putrescine can be further catabolized by diamine oxidases (DAO), which is a copper containing protein catalyzing oxidation of putrescine to 4-aminobutanal, NH_3_, and H_2_O_2_ (Cona et al., 2006). The resulting aldehyde of both PAO and DAO reaction is then further converted to γ-aminobutyric acid (GABA) via pyrroline. The genome of *A. thaliana* contains five genes encoding PAOs (Tavladoraki et al., 2006), which are all expressed in flowers and stamens and accept PAs with more than two amino groups as a substrate (Takahashi et al., 2010). However, in our study at least at the transcript level no change in the PAOs in wild type compared to *sht* is evident. Currently, there is no indication that accumulating PAs in *sht* anthers are removed by enzymatic deamination. With respect to thermospermine, the high transcript levels in wild type and *sht* lines compared to the low amounts of this PA (if our preliminary identification is correct) appear surprising. This may suggest that re-direction of the pathway toward thermospermine biosynthesis could be relevant under yet unknown conditions. The unchanged transcript levels of the second HCAA-transferase SDT and virtual absence of the SCT in wild type and *sht* mutants are consistent with the irrelevance of these enzymes in *Arabidopsis* stamens, as compared to seeds or roots (Luo et al., 2009), but illustrates the organ specificity and apparent relevance for tris-conjugated spermidine conjugates for pollen integrity.

In conclusion, this sensitive method using FMOC-Cl as derivatizing reagent for the determination of PAs by HPLC will allow closely monitoring PA levels even when only small amounts of plant material are available. The method can easily adapted to a high-throughput procedure useful for a screening program to correlate PA levels with various abiotic and biotic stress conditions and monitor PA metabolism with high sensitivity (Alcázar et al., 2010). Whether PA homeostasis and the cross-talk with the phenylpropanoid metabolism is maintained in stamens and specifically in the tapetum by translational control of *SAMDC1*, by oxidation of redundant PAs, or by transport into other organs can be revealed by monitoring the metabolic fluxes in the tapetum during pollen maturation, by elucidation of transport and oxidation mechanisms, and analyzing co-expression data of relevant genes.

## Conflict of Interest Statement

The authors declare that the research was conducted in the absence of any commercial or financial relationships that could be construed as a potential conflict of interest.
